# Outcome of SARS-CoV-2-Infected Polish Patients with Chronic Lymphocytic Leukemia

**DOI:** 10.3390/cancers14030558

**Published:** 2022-01-22

**Authors:** Bartosz Puła, Katarzyna Pruszczyk, Ewa Pietrusza, Marta Morawska, Weronika Piszczek, Elżbieta Kalicińska, Agnieszka Szeremet, Jagoda Tryc-Szponder, Ewa Wąsik-Szczepanek, Joanna Drozd-Sokołowska, Helena Krzemień, Aleksandra Rejus, Małgorzata Gajewska, Kamil Wiśniewski, Maciej Wysocki, Alan Majeranowski, Ewa Paszkiewicz-Kozik, Paweł Steckiewicz, Łukasz Szukalski, Łukasz Bołkun, Monika Długosz-Danecka, Krzysztof Giannopoulos, Krzysztof Jamroziak, Ewa Lech-Marańda, Iwona Hus

**Affiliations:** 1 Department of Hematology, Institute of Hematology and Transfusion Medicine, 02-776 Warsaw, Poland; bpula@ihit.waw.pl (B.P.); kpruszczyk@ihit.waw.pl (K.P.); kwisniewski@ihit.waw.pl (K.W.); mwysocki@ihit.waw.pl (M.W.); emaranda@ihit.waw.pl (E.L.-M.); 2 Maria Sklodowska-Curie National Research Institute of Oncology, 31-115 Cracow, Poland; ewapie3@wp.pl (E.P.); monika.dlugosz-danecka@lymphoma.edu.pl (M.D.-D.); 3 Experimental Hematooncology Department, Medical University, 20-400 Lublin, Poland; martamorawska@umlub.pl (M.M.); krzysztof.giannopoulos@umlub.pl (K.G.); 4 Department of Hematology, St. John’s Cancer Center, 20-090 Lublin, Poland; 5 Department of Hematology, Copernicus Hospital, 87-100 Torun, Poland; weronika.piszczek@med.torun.pl; 6 Department and Clinic of Hematology, Blood Neoplasms and Bone Marrow Transplantation, Wroclaw Medical University, 52-007 Wroclaw, Poland; elzbieta.kalicinska@umw.edu.pl (E.K.); agnieszka.szeremet@umw.edu.pl (A.S.); 7 Department of Hematology and Bone Marrow Transplantation, Poznan University of Medical Sciences, 60-569 Poznan, Poland; jagoda.tryc@skpp.edu.pl; 8 Department of Hematooncology and Bone Marrow Transplantation, Medical University, 20-400 Lublin, Poland; ewawsz@poczta.onet.pl; 9 Department of Hematology, Transplantation and Internal Medicine, Medical University, 02-097 Warsaw, Poland; joanna.drozd-sokolowska@wum.edu.pl (J.D.-S.); kjamroziak@wum.edu.pl (K.J.); 10 Department of Hematology and Bone Marrow Transplantation, Medical School of Silesia, Silesian Medical University, 40-032 Katowice, Poland; hkrzemien@gmail.com; 11 Department of Hematology, Regional Clinical Hospital No. 1, 35-001 Rzeszow, Poland; aleksandra.rejus@gmail.com; 12 Department of Internal Medicine and Hematology, Military Institute of Medicine, 04-141 Warsaw, Poland; mgajewska@wim.mil.pl; 13 Intercollegiate Faculty of Biotechnology of the University of Gdansk and the Medical University of Gdansk, 80-307 Gdansk, Poland; alan.majeranowski@gumed.edu.pl; 14 Department of Hematology and Transplantology, Medical University, 80-211 Gdansk, Poland; 15 Department of Lymphoid Malignancies, Maria Sklodowska-Curie National Research Institute of Oncology, 00-001 Warsaw, Poland; ewa.paszkiewicz-kozik@pib-nio.pl; 16 Collegium Medicum, Jan Kochanowski University, 25-317 Kielce, Poland; pawel.steckiewicz@onkol.kielce.pl; 17 Department of Hematology, Holy Cross Cancer Center, 25-734 Kielce, Poland; 18 Department of Hematology, Collegium Medicum in Bydgoszcz, Nicolaus Copernicus University in Torun, 85-168 Bydgoszcz, Poland; lukasz.szukalski@cm.umk.pl; 19 Department of Hematology, Medical University, 15-276 Bialystok, Poland; lukasz.bolkun@umb.edu.pl

**Keywords:** chronic lymphocytic leukemia, SARS-CoV-2, infection, prognosis, COVID-19

## Abstract

**Simple Summary:**

The severe acute respiratory syndrome coronavirus (SARS-CoV-2) has become the cause of a worldwide pandemic, and its clinical infection course in patients with hematological malignancies may be severe. Chronic lymphocytic leukemia (CLL) patients are among this group, and CLL-directed therapies are discussed as potential COVID-19-severity modifying agents. So far, the published data and clinical experience in treatment of COVID-19 patients with CLL are still scarce. Therefore, we aimed at retrospectively analyzing factors associated with SARS-CoV-2 infection course in patients with CLL.

**Abstract:**

Background. The severe acute respiratory syndrome coronavirus (SARS-CoV-2) has become the cause of a worldwide pandemic, and its clinical infection course in patients with hematological malignancies may be severe. Methods. We performed a retrospective study on 188 chronic lymphocytic leukemia patients (CLL) with COVID-19 infection. Results. At the time of infection 51 patients (27.1%) were treated with Bruton tyrosine kinase inhibitor (BTKi), 46 (24.5%) with anti-CD20 antibodies while 37 patients (19.7%) received venetoclax. In total, 111 patients (59.0%) required hospitalization and 50 patients (26.5%) died due to COVID-19. Patients with poor performance status (ECOG >1; *p* = 0.02), advanced age (>65 years; *p* = 0.04), low hemoglobin concentration (≤10 g/dl; *p* = 0.0001), low platelets (<100 × 109/L; *p* = 0.003), and elevated lactate dehydrogenase level (LDH; *p* = 0.014) had an increased risk of death due to COVID-19. Neither CLL treatment status (treatment naïve vs. treated) nor the type of CLL-directed treatment had impact on the SARS-CoV-2 related risk of death. The multivariate survival analysis showed that advanced age (*p* = 0.009) and low platelet count (*p* = 0.0001) were associated with significantly shorter patients’ overall survival. Conclusions. SARS-CoV-2 infection in CLL patients is associated with poor outcome regardless of administered CLL-directed treatment.

## 1. Introduction

Severe acute respiratory syndrome coronavirus 2 (SARS-CoV-2), responsible for coronavirus disease 19 (COVID-19), has led to a significant morbidity increase worldwide since its outbreak in December 2019 in Wuhan, China [1]. The severity of the disease can vary between particular patients, however advanced age, cardiovascular comorbidities and history of cancer were shown to be associated with worse outcome [2,3,4,5]. Chronic lymphocytic leukemia (CLL) is the most common leukemia, with a median age at diagnosis reaching 72 years, and often patients at diagnosis are characterized by the presence of other comorbidities potentially affecting treatment possibilities and outcome [6]. CLL is characterized by defects of both adoptive and innate immune response, and the administered antileukemic treatment further strengthens immune defects, affecting elimination of SARS-CoV-2 leading to poor patient outcome [7,8]. A recently performed meta-analysis showed a high case fatality rate (CFR) of 34% in 3240 adult, mainly hospitalized patients with hematological malignancies [9]. Indeed, published reports indicate a closely related CFR in CLL patients which ranges from 27.3% to 35% depending on the study, rate of hospitalization (60–78%) and time from the beginning of the COVID-19 pandemic [10,11,12,13]. The observational study of Roeker et al. showed that some improvement in the management of COVID-19 patients had been made, as the CFR rate dropped from 35% at the beginning of the pandemic to 11% at later months [12].

Despite this fact, the morbidity of CLL patients infected with the SARS-CoV-2 virus remains high, and further progress is needed. Although several agents have been approved for COVID-19 treatment and dexamethasone showed a significant impact in patients requiring oxygen supplementation on overall survival (OS), the number of hematological patients in these studies was low and does not allow for translating these findings to the field of immunocompromised patients [14,15,16,17]. Interestingly, analysis of COVID-19-directed therapies in CLL-hospitalized patients revealed that remdesivir treatment and convalescent fresh frozen plasma (CCP) transfusion reduced the risk of death, whereas dexamethasone and hydrochloroquine administration increased it [12].

Despite the growing number of agents being approved for the treatment of COVID-19, this disease still poses a serious clinical problem in CLL patients. In this paper, we analyze the outcome of SARS-CoV-2 infection in 188 CLL patients based on the data collected within a retrospective study conducted by the Polish Adult Leukemia Study Group (PALG).

## 2. Materials and Methods

### 2.1. Patients

The study was conducted in accordance with the provisions of the Declaration of Helsinki and the International Conference on Harmonization Guidelines for Good Clinical Practice. The data were acquired retrospectively by the Polish Adult Leukemia Group (PALG) sites by identifying patients from their local registries. CLL patients with SARS-CoV-2 infection confirmed by a positive test result using rapid antigen detection or real-time polymerase chain reaction (PCR) test were included in the analysis. Treatment indications and response assessments were based on the 2018 International Workshop on Chronic Lymphocytic Leukemia (IWCLL) criteria and performed by the enrolling physicians. 

Data were collected between 25 March 2020 and 7 May 2021. Vaccines against SARS-CoV-2 in Poland became available at the end of December 2020, first for the medical staff, then for the subsequent age groups, starting from octogenarians. SARS-CoV-2 vaccination became available for oncological patients only in March 2021, which means that at the time of analysis, most patients were not immunized against SARS-CoV-2. 

### 2.2. Statistical Analysis

The data were analyzed in Statistica 13.3 (Dell Inc., StatSoftPolska Sp. z o.o., Cracow, Poland), Graph Pad Prism 9 (La Jolla, CA, USA) and SAS software (SAS Institute Inc., Cary, NC, USA). For univariate analysis Kruskal–Wallis, Mann–Whitney U, Fisher’s exact and chi-squared tests were employed. The overall survival (OS) was defined as the time from positive SARS-CoV-2 test to death and was analyzed with a log-rank test. Multivariate analysis of OS was performed using the Cox proportional hazard model. The level of statistical significance was set at *p* < 0.05.

## 3. Results

### 3.1. Patients’ Characteristics

The study group included 188 CLL patients. The median age of the patients at COVID-19 diagnosis was 67.9 years (range 36–87), and 70 (37.2%) were men (Table 1). COVID-19 diagnosis was made in 173 patients (92.0%) on the basis of a positive PCR test, whereas in 16 patients (8.5%) it was based on the positive result of the antigen test. The Median Eastern Cooperative Study Group (ECOG) score was 1 (range 0–4). At the time of SARS-CoV-2 infection, 29 (15.4%) patients were treatment-naïve (TN), 41 (21.8%) had ended treatment, whereas 118 (62.8%) were undergoing an active phase of CLL therapy. The median number of lines of previously administered treatment regimens was 1 (range 0–7), whereas 24 (12.8%) patients received four or more treatment lines. At the time of infection, 51 patients (27.1%) were being treated with Bruton tyrosine kinase inhibitor (BTKi), 46 (24.5%) with anti-CD20 antibodies, while 37 patients (19.7%) were receiving venetoclax therapy. The median follow-up time was 72 days (range 0–334).

### 3.2. Survival Analysis of the Whole Study Cohort

In the study group, 50 patients (26.5%) died due to COVID-19 disease. In the nonhospitalized group, 7 out of 77 patients (9.1%) died. Patients with poor performance status (ECOG > 1; *p* = 0.02), advanced age (>65 years; *p* = 0.04), low hemoglobin concentration (≤10 g/dL; *p* = 0.0001), low platelet count (<100 × 109/L; *p* = 0.003), and elevated lactate dehydrogenase level (LDH; *p* = 0.014) were at the increased risk of death due to COVID-19 (Supplementary Table S1). The multivariate analysis revealed that independent factors associated with risk of death due to SARS-CoV-2 infection and its complications included advanced age (*p* = 0.02), low hemoglobin concentration (*p* = 0.0019) and platelet level (*p* = 0.004).

**Figure 1:**
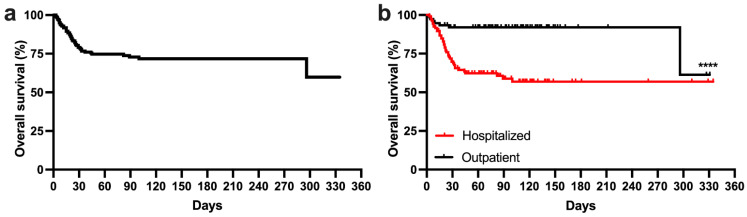
Kaplan–Meier survival curves of the whole (**a**) and hospitalized (**b**) patient cohorts. **** *p* < 0.0001 (Log-rank test).

**Figure 2:**
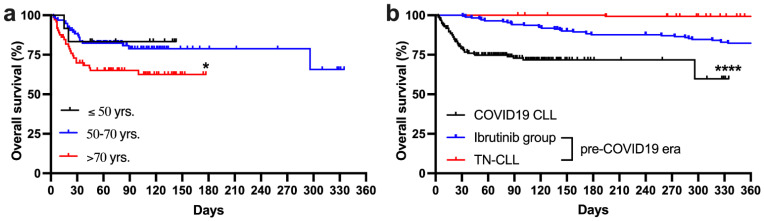
Kaplan–Meier survival curves of the whole cohort in particular age groups (**a**) and in comparison to treatment-naïve chronic lymphocytic leukemia patients (TN–CLL) and observational relapse and refractory chronic lymphocytic leukemia treated with ibrutinib (ibrutinib group) patients form the pre-COVID-19 time (**b**). Data were cut to 360 days on the time axis. * *p* < 0.05; **** *p* < 0.0001 (Log-rank test).

Median OS of the whole study cohort was not reached (Figure 1a). The univariate survival analysis showed that advanced age (>65 years; HR 2.07; 95% CI 1.17–3.45; *p* = 0.04), poor performance status (ECOG > 1; HR 2.13; 95% CI 1.07–4.2; *p* = 0.007), low hemoglobin level (Hb ≤ 10 g/dL; HR 2.82; 95% CI 1.42–5.6; *p* = 0.0005), low platelet count (PLT < 100 × 10^9^/L; HR 2.69; 95% CI 1.36–5.29; *p* = 0.0012), and elevated LDH level (HR 1.9; 95% CI 1.05–3.52; *p* = 0.008) were associated with significantly shorter overall survival (Supplementary Table S2 and Figure S1). Patients aged older than 70 years were identified to have the worst outcome (*p* < 0.03) (Figure 2a). Multivariate Cox regression hazard analysis showed that only advanced age ((HR 2.27, 95% CI 1.22–4.17) and low platelet count (HR 3.04, 95% CI 1.72–5.36, *p* = 0.0001) were independent factors for shorter OS. To assess the impact of COVID-19 on patients, we compared the current cohort of patients to two observational groups stemming from pre-COVID-19 times. The first cohort comprised 138 treatment-naïve CLL patients diagnosed in the years 2010–2014 at the Institute of Hematology and Transfusion Medicine (Supplementary Table S3). The second historical cohort included 171 patients with relapse-refractory CLL treated with ibrutinib monotherapy in a compassionate drug-use program collected within the observational study of Polish Adult Leukemia Study Group (Figure 2b) [18]. Analysis of the Kaplan–Meier survival curves revealed the significant impact of COVID-19 infection on patient overall survival (*p* < 0.0001). The 180-day survival rate in the analyzed COVID-19 cohort was 71.6%, whereas in the TN-CLL and ibrutinib treated groups it was 100% and 87.7%, respectively.

### 3.3. Risk Factors for Hospitalization

In the analyzed cohort, 111 patients (59.0%) required hospitalization. Poor ECOG performance status (*p* < 0.001), hemoglobin levels (Hb ≤ 10 g/dL; *p* < 0.0001), low platelet count (PLT < 100 × 10^9^/L; *p* = 0.012), elevated LDH level (*p* = 0.09), advanced Binet stage at diagnosis (*p* = 0.048) and treatment with anti-CD20-directed antibodies (*p* = 0.01) were associated with the need of hospitalization due to SARS-CoV-2 infection (Supplementary Table S4). The multivariate analysis revealed that the independent factors associated with the risk of hospitalization due to SARS-CoV-2 infection and its complications included the presence of 17p deletion (*p* = 0.042), anti-CD20 antibody treatment (*p* = 0.02), low hemoglobin (*p* = 0.008), and platelet (*p* = 0.004) levels and elevated LDH level (*p* = 0.0023).

### 3.4. Analysis of the Hospitalized Subgroup

Data of hospitalized patients are summarized in Table 1. Overall, 25 (22.5%) patients were hospitalized in the intensive care unit (ICU). The median time of hospitalization in hematological/internal ward was 14 days (range 1–150), whereas it was 8.5 days (range 1–41) in the ICU. The median follow-up time in the hospitalized patients was 45 days (0–334). At the time of data acquisition, 32 (28.8%) patients (3 patients in the ICU) were still hospitalized. The hospitalized patients were treated according to their respective center guidelines. Overall, 99 (89.1%) patients required oxygen supplementation. High-flow nasal oxygen therapy (HFNOT) and mechanical ventilation were used in 35 (31.8%) and 22 (20.0%) patients, respectively. Among the hospitalized patients, 93 (84.5%) received antibiotics, 77 (69.4%) received dexamethasone, low-molecular weight heparin (LMWH) was given to 71 (64.5%) patients, 52 (46.8%) received COVID-19 convalescent fresh frozen plasma (CCP), whereas 39 (35%) patients underwent remdesivir therapy. Out of the patient-dependent factors known at the time of COVID-19 diagnosis, only low platelet count (*p* = 0.02) and low hemoglobin level (*p* = 0.02) were associated with an increased risk of death due to COVID-19 (Table 2). In the multivariate analysis, the risk of death was associated with low hemoglobin level (*p* = 0.02) and lower platelet count (*p* = 0.009).

In the hospitalized group, 43 (38.7%) patients died. Median overall survival in the hospitalized group cohort was not reached (Figure 1b). Survival comparison of hospitalized patients and those treated as outpatients revealed significantly shorter OS (HR 4.94, 95% CI 2.79–8.69; *p* < 0.0001). The univariate survival analysis showed that only low hemoglobin and platelet counts were associated with worse patient survival outcome (HR 2.0, 95% CI 1.05–3.8, *p* = 0.04 and HR 2.37, 95% CI 1.2–4.67, *p* = 0.004, respectively, Table 3; Supplementary Figure S2). Multivariate survival analysis showed that advanced age (HR 2.13, 95% CI 1.09–4.17) and low platelet count (HR 2.85, 95% CI 1.53–5.31; *p* = 0.0009) were associated with significantly shorter OS.

## 4. Discussion

In this paper, we confirm that COVID-19 poses a serious threat to CLL patients with a CFR of 26.5% observed in the whole cohort. An even more dismal outcome was noted in patients requiring hospitalization due to COVID-19 infection in whom CFR rate reached 38.7%. Our data are comparable in this case to other so-far published national and international multicenter studies, where CFRs ranged from 27.3% to 35% [10,11,12,13]. The data gathered in this study stem from the second and third waves of the COVID-19 pandemic in Poland, when experience in the treatment of COVID-19 disease was very limited and the SARS-CoV-2 vaccination program was at the earliest phase of its development. Therefore, the data generated in our observational study should be compared mostly to the first observational COVID-19 study reports, as the development of new SARS-CoV-2-directed agents and gains in clinical experience based on the observations of Roeker et al. led to outcome improvement in CLL patients by reducing the CFR from 35% to 11% [12]. 

In the analyzed cohort, 59% of patients required hospitalization, and overall 99 (89.1%) patients required oxygen supplementation, whereas mechanical ventilation was necessary in 22 (20.0%). Although we did observe a slightly lower number of hospitalized patients in our cohort compared to other studies, the severity of the infection in the hospitalized patients seems to be comparable to the data presented in other observational studies [10,11,12,13]. It is noteworthy that some studies underline that introduction of dexamethasone, remdesivir and gains in clinical experience helped to reduce the CFR, as shown in the studies of Roeker et al. and Blixt et al. [12,19]. Interestingly, in the study of Roeker et al., in the later phase of the pandemic a reduced number of hospitalizations for CLL patients with COVID-19 was observed (85% vs. 55%) as well as ICU admissions (32% vs. 15%), and this also could have the beneficial effect on the observed lower mortality in the later phase of the pandemic [12]. In contrast, in the study of Blixt et al., no significant difference in the hospitalization rates was noted (86% vs. 71%). However, the observed reduction in deaths from 32% to 18% could be related to the increasing clinical experience and broader use of remdesivir and dexamethasone in the later period of the pandemic in Sweden (5% to 41% and 47% to 78%) [19]. On the other hand, the largest analysis of CLL patients with COVID-19 did not show any improvement between outcomes of subsequent waves of SARS-CoV-2 infection [10]. Considering the variable drug availability, heterogenous populations, and differences in local treatment protocols, a strict comparison of patient outcomes between these studies is not possible. 

We observed the increased risk of death in patients with advanced age. In our study, we confirmed that advanced age is a significant factor for poor outcomes with COVID-19, which corroborates the results of other observational studies [3,4,10,13]. Interestingly we also noted that low hemoglobin and platelet levels were associated with the patient’s increased risk of death, which to our knowledge has not been reported so far. In our opinion, this observation may reflect CLL disease activity and severity, and therefore could additionally increase patients’ risk of death. In the whole cohort, the median OS was not reached; however, in terms of the analyzed factors, only advanced age and low platelet count were associated with shorter OS. Although the impact of advanced patient age on shorter OS was well-described in the previous studies, the impact of low platelet levels should be regarded with caution due to limitations in the observational nature of our study [3,4,10,13].

Low hemoglobin and platelet levels, in addition to the presence of 17p deletion, elevated LDH and anti-CD20 antibody treatment, were factors significantly associated with the need for hospitalization, and therefore they potentially contribute to the increased mortality, mirroring the severity of COVID-19. Although, the use of anti-CD20 antibodies was not shown to impact the patient risk of death or OS in the multivariate analysis, our data indicate that the use of such agents may lead to a more severe COVID-19 clinical course. Our data partially confirm the observation from the large multicenter study performed in 941 CLL patients, which showed that the use of anti-CD20 antibodies alone or in combination led to the shorter OS when compared to untreated patients [10]. However, we did not observe any impact of the previous administration of CLL-directed treatment or the impact of venetoclax or BTK inhibitors on the patient risk of hospitalization. 

Interestingly we found that low platelet counts were associated with poor outcome in the whole and hospitalized patient cohort. Platelets have been discussed to modify course of the disease in various cancers, including CLL [20,21]. It was shown that thrombocytopenia was associated with poor outcome of COVID-19 patients in intensive care units [22]. These results are also corroborated by two independently performed meta-analyses which point to poor outcomes in patients with low platelet counts [23,24]. We also showed that thrombocytopenia was associated with increased risk of hospitalization in CLL COVID-19 patients; however, a recently published report does not corroborate our finding as platelet count did not affect the SARS-CoV-2 infection outcome in hospitalized patients [25].

In this study, we could not address the issue of COVID-19-directed treatment due to the retrospective nature of the study and data heterogeneity. The roles of remdesivir and CCP administration have shown beneficial effects in some studies, however their efficacy in CLL patients is questionable [1,12,17,26,27]. In the hospitalized cohort, we did not observe any impact of CLL treatment status history or impact of potential agents (anti-CD20 antibodies, BTKi and venetoclax), although some studies suggest the protective role of BTK inhibitors or increased severity during venetoclax therapy [11,13,28,29]. It must be stressed that the data presented in this analysis have several limitations, and assessment of the effectivity of particular agents, COVID-19- or CLL-directed, was not the primary aim of our observational study. Furthermore, the treatment of COVID-19 was administered in accordance with local guidelines, not within clinical trials. In addition, due to the time-dependent availability, particular agents (e.g., remdesivir or CCP) could not have been administered in a timely manner dependent on the disease severity.

Besides the abovementioned limitations due to testing policy in Poland, in the second and third waves of COVID-19 we potentially could have missed some asymptomatic patients. Another limitation of this study is the lack of detailed assessment of patient comorbidities. In our analysis, we assessed patient performance on the ECOG scale, however, proper assessment of patient disease burden based on the type of comorbidity or its precise definition severity using the Cumulative Illness Rating Scale (CIRS) was not possible. We acknowledge that these data would be of utmost importance in assessing factors associated with COVID-19 outcome.

## 5. Conclusions

In view of the presented results, SARS-CoV-2 infection in patients with CLL is associated with poor outcome regardless of administered CLL-directed treatment. Due to the retrospective nature of the study and collection of data outside clinical trial regulations we could not assess COVID-19-directed treatments. This issue should be addressed within clinical trials to assess the potential clinical benefit of COVID-19-directed therapies in immunocompromised CLL patients.

## Figures and Tables

**Table 1:** Clinicopathological characteristics of all analyzed patients and patients requiring hospitalization.

	All Patients	Hospitalized Patients
**Age (median; range)**	68 (37–87)	69 (37–87)
**Parameter**	N (%)	N (%)
**Sex**		
Men	119 (63.3%)	70 (63.1%)
Women	69 (36.7%)	41 (36.9%)
**Rai stage**		
0	13 (6.9%)	8 (7.2%)
1	40 (21.3%)	23 (20.7%)
2	69 (36.7%)	36 (32.4%)
3	28 (14.9%)	15 (13.5%)
4	32 (17%)	23 (20.7%)
NA	6 (3.2%)	6 (5.4%)
**Binet stage**		
A	45 (23.9%)	19 (17.1%)
B	70 (37.2%)	40 (36%)
C	29 (15.4%)	20 (18%)
NA	44 (23.4%)	32 (28.8%)
**ECOG**		
2–4	46 (24.5%)	37 (33.3%)
0–1	136 (72.3%)	71 (64%)
NA	6 (3.2%)	3 (2.7%)
**WBC [×10^9^/L]**		
≤25	132 (70.2%)	77 (69.4%)
>25	56 (29.8%)	34 (30.6%)
**Hemoglobin [g/dL]**		
≤10	44 (23.4%)	39 (35.1%)
>10	143 (76%)	71 (64%)
NA	1 (0.5%)	1 (0.9%)
**Platelets [×10^9^/L]**		
≤100	51 (27.1%)	39 (35.1%)
>100	137 (72.9%)	72 (64.9%)
**Lactate dehydrogenase**		
Elevated	71 (37.8%)	50 (45%)
Normal range	90 (47.9%)	43 (38.7%)
NA	27 (14.4%)	18 (16.2%)
**BMI**		
<18.5	1 (0.5%)	1 (0.9%)
18.5–25	48 (25.5%)	26 (23.4%)
25–30	87 (46.3%)	50 (45%)
>30	36 (19.1%)	22 (19.8%)
NA	16 (8.5%)	12 (10.8%)
**Creatinine [mg/dL]**		
>1.3	19 (10.1%)	14 (12.6%)
≤1.3	163 (86.7%)	95 (85.6%)
NA	6 (3.2%)	2 (1.8%)
**Deletion 17p**		
Yes	23 (12.2%)	71 (64%)
No	126 (67%)	14 (12.6%)
NA	39 (20.7%)	26 (23.4%)
***TP53* mutation**		
Yes	14 (7.4%)	59 (53.1%)
No	106 (56.4%)	10 (9%)
NA	68 (36.2%)	42 (37.8%)
**Deletion 11q23**		
Yes	21 (11.2%)	56 (50.5%)
No	100 (53.2%)	14 (12.6%)
NA	67 (35.6%)	41 (36.9%)
**CLL treatment status**		
Treatment-naive	29 (15.4%)	15 (13.5%)
During treatment	117 (62.2%)	74 (66.7%)
After treatment	41 (21.8%)	22 (19.8%)
NA	1 (0.5%)	0 (0.0%)
**Lines of previous therapy**		
≥4	23 (12.2%)	17 (15.3%)
0–3	164 (87.2%)	94 (84.7%)
NA	1 (0.5%)	0 (0.0%)
**BTKi treatment**		
No	137 (72.9%)	88 (79.3%)
Yes	51 (27.1%)	23 (20.7%)
**Venetoclax treatment**		
Yes	50 (26.6%)	23 (20.7%)
No	138 (73.4%)	88 (79.3%)
**Anti-CD20 treatment**		
Yes	46 (24.5%)	34 (30.6%)
No	142 (75.5%)	77 (69.4%)

BMI—body mass index; BTKi—Brutons tyrosine kinase inhibitor; ECOG—Eastern Cooperative Oncology Group; NA—not available; WBC—white blood count.

**Table 2:** Factors associated with increased risk of death in the hospitalized patients.

	No		Yes		
**Gender**	N	(%)	N	(%)	*p*-value
Men	43	61.43%	27	38.57%	0.99
Women	25	60.98%	16	39.02%	
**Age**					
>65	39	56.52%	30	43.48%	0.23
≤65	29	69.05%	13	30.95%	
**Rai Stage**					
0	3	37.50%	5	62.50%	0.43
1	16	69.57%	7	30.43%	
2	21	58.33%	15	41.67%	
3	11	73.33%	4	26.67%	
4	13	56.52%	10	43.48%	
**Binet stage**					
A	12	63.16%	7	36.84%	0.87
B	24	60.00%	16	40.00%	
C	11	55.00%	9	45.00%	
**ECOG**					
2–4	47	66.20%	24	33.80%	0.15
0–1	19	51.35%	18	48.65%	
**WBC [** **×10^9^/L]**					
≤25	48	62.34%	29	37.66%	0.83
>25	20	58.82%	14	41.18%	
**Hemoglobin [g/dL]**					
≤10	18	46.15%	21	53.85%	0.02
>10	49	69.01%	22	30.99%	
**Platelets [** **×10^9^/L]**					
≤100	18	46.15%	21	53.85%	0.02
>100	50	69.44%	22	30.56%	
**Lactate dehydrogenase**					
Elevated	26	52.00%	24	48.00%	0.21
Normal range	28	65.12%	15	34.88%	
**BMI**					
<18.5	1	100.00%	0	0.00%	0.74
18.6–24.9	17	65.38%	9	34.62%	
25–30	31	62.00%	19	38.00%	
>30	12	54.55%	10	45.45%	
**Creatinine [mg/dL]**					
>1.3	8	57.14%	6	42.86%	0.6
≤1.3	59	62.11%	36	37.89%	
**Deletion 17p**					
Yes	10	71.43%	4	28.57%	0.55
No	43	60.56%	28	39.44%	
**Deletion 11q23**					
Yes	9	64.29%	5	35.71%	0.99
No	35	62.50%	21	37.50%	
***TP53* mutation**					
Yes	5	17.86%	23	82.14%	0.73
No	5	50.00%	5	50.00%	
**CLL treatment status**					
Treatment naive	8	53.33%	7	46.67%	0.79
After	14	63.64%	8	36.36%	
During	46	62.16%	28	37.84%	
**Lines of previous therapy**					
≥4	10	58.82%	7	41.18%	0.99
0–3	58	61.70%	36	38.30%	
**BTKi treatment**					
No	52	61.18%	33	38.82%	0.99
Yes	55	62.50%	33	37.50%	
**Venetoclax treatment**					
Yes	13	56.52%	10	43.48%	0.64
No	112	73.68%	40	26.32%	
**Anti-CD20 treatment**					
Yes	20	58.82%	14	41.18%	0.83
No	48	62.34%	29	37.66%	

**Table 3:** Overall survival of hospitalized patients.

	No. of Patients	OS [Days]	95% CI	HR	95% CI	*p*-Value
**Gender**						
Men	70	nr	45–nr	1.02	0.55–1.91	0.76
Women	41	nr	33–nr			
**Age**						
>65	69	nr	29–nr	1.71	0.92–3.18	0.14
≤65	42	nr	89–nr			
**Rai Stage**						
0	8	29	15–nr			0.35
1	23	nr	21–nr			
2	36	nr	33–nr			
3	15	nr	44–nr			
4	23	nr	23–nr			
**Binet stage**						
A	19	nr	20–nr			0.89
B	40	nr	82–nr			
C	20	nr	19–nr			
**ECOG**						
2–4	37	nr	33–nr	1.55	0.8–3.0	0.59
0–1	71	nr	89–nr			
**WBC [×10^9^/L]**						
≤25	77	nr	31–nr	0.97	0.5–1.85	0.92
>25	34	nr	31–nr			
**Hemoglobin [g/dL]**						
≤10	39	44	29–nr	2.0	1.05–3.8	0.04
>10	71	nr	nr			
**Platelets [×10^9^/L]**						
≤100	39	31	19–nr	2.37	1.2–4.67	0.004
>100	72	nr	nr			
**Lactate dehydrogenase**						
Elevated	50	100	22–nr	1.3	0.68–2.49	0.28
Normal range	43	nr	33–nr			
**BMI**						
<18.5	1	nr	nr			0.8
18.5–25	26	nr	44–nr			
25–30	50	nr	29–nr			
>30	22	nr	25–nr			
**Creatinine [mg/dL]**						
>1.3	14	100	26–nr	1.34	0.47–3.83	0.32
≤1.3	95	nr	82–nr			
**Deletion 17p**						
No	71	nr	45–nr	1.35	0.53–3.48	0.59
Yes	14	nr	8–nr			
**Deletion 11q23**						
Yes	14	nr	15–nr	1.1	0.4–3.04	0.92
No	56	nr	82–nr			
***TP53* mutation**						
Yes	10	nr	3–nr	0.65	0.21–2.0	0.42
No	59	nr	45–nr			
**CLL treatment status**						
Treatment naive	15	37	20–nr			0.64
After	22	nr	33–nr			
During	74	nr	82–nr			
**Lines of previous therapy**						
≥4	17	nr	21–nr	1.01	0.45–2.29	0.91
0–3	94	nr	45–nr			
**BTKi treatment**						
No	85	370	45–nr	1.07	0.52–2.21	0.99
Yes	26	nr	21–nr			
**Venetoclax treatment**						
Yes	23	100	27–nr	1.2	0.57–2.54	0.73
No	88	nr	82–nr			
**Anti-CD20 treatment**						
Yes	32	nr	23–nr	1.34	0.68–2.66	0.5
No	77	nr	82–nr			

## Data Availability

Study data are available upon request to the correspondence author.

## Supplementary Materials

The following are available online at https://www.mdpi.com/article/10.3390/cancers14030558/s1, Table S1: Association of analyzed factors with risk of death; Table S2: Overall survival of all analyzed patients; Table S3: Characterization of treatment-naïve chronic lymphocytic leukemia patients diagnosed in the years 2010–2014 at the Institute of Hematology and Transfusion Medicine; Table S4: Association of analyzed factors with risk of hospitalization; Figure S1: Kaplan–Meier overall survival curves of the whole analyzed cohort in relation to clinicopathological parameters; Figure S2: Kaplan–Meier overall survival curves of hospitalized patients in relation to clinicopathological parameters.
