# Supplementary material for: Outcome of SARS-CoV-2-Infected Polish Patients with Chronic Lymphocytic Leukemia
Source: Cancers (Basel). 2022 Jan 22;14(3):558. doi: 10.3390/cancers14030558 (PMC8833688; doi:10.3390/cancers14030558)

Table S1. Association of analyzed factors with risk of death.

	No		Yes		
**Gender**	N	(%)	N	(%)	p-value
Men	87	73.11%	32	26.89%	0.9999
Women	51	73.91%	18	26.09%	
**Age**					
> 65	76	67.86%	36	32.14%	0.04
≤ 65	62	81.58%	14	18.42%	
**Rai Stage**					
0	8	61.54%	5	38.46%	0.58
1	32	80,00%	8	20,00%	
2	52	75.36%	17	24.64%	
3	21	75,00%	7	25,00%	
4	21	65.63%	11	34.38%	
**Binet stage**					
A	37	82.22%	8	17.78%	0.15
B	53	75.71%	17	24.29%	
C	18	62.07%	11	37.93%	
**ECOG**					
2-4	27	58.7%	19	41.3%	0.02
0-1	106	77.94%	30	22.06%	
**WBC [×10^9/L]**					
≤ 25	96	72.73%	36	27.27%	0.86
> 25	42	75,00%	14	25,00%	
**Hemoglobin [g/dL]**					
≤ 10	22	50,00%	22	50,00%	0.0001
> 10	115	80.42%	28	19.58%	
**Platelets [×10^9/L]**					
≤ 100	29	56.86%	22	43.14%	0.003
> 100	109	79.56%	28	20.44%	
**Lactate dehydrogenase**					
Elevated	44	61.97%	27	38.03%	0.014
Normal range	72	80.00%	18	20.00%	
**BMI**					
<18.5	1	100,00%	0	0,00%	0.45
18.6 – 24.9	37	77.08%	11	22.92%	
25-30	66	75.86%	21	24.14%	
>30	23	63.89%	13	36.11%	
**Creatinine [mg/dL]**					
> 1.3	13	68.42%	6	31.58%	0.6
≤ 1.3	120	73.62%	43	26.38%	
**Deletion 17p**					
No	94	74.6%	32	25.4%	0.8
Yes	18	78.26%	5	21.74%	
**Deletion 11q23**					
Yes	15	71.43%	6	28.57%	0.78

No	76	76.00%	24	24.00%	
***TP53* mutation**					
Yes	8	57.14%	6	42.86%	0.21
No	78	73.58%	28	26.42%	
**CLL treatment status**					
Treatment naive	22	75.86%	7	24.14%	0.84
After	31	75.61%	10	24.39%	
During	84	71.79%	33	28.21%	
**Lines of previous therapy**					
≥ 4	16	69.57%	7	30.43%	0.63
0 – 3	121	73.78%	43	26.22%	
**BTKi treatment**					
No	100	72.99%	37	27.01%	0.5
Yes	38	74.51%	13	25.49%	
**Venetoclax treatment**					
Yes	26	72.22%	10	27.78%	0.5
No	112	73.68%	40	26.32%	
**Anti-CD20 treatment**					
Yes	31	67.39%	15	32.61%	0.34
No	107	75.35%	35	24.65%	

BMI – body mass index; BTKi – Brutons tyrosine kinase inhibitor; ECOG – Eastern Cooperative Oncology Group; NA – not availabe; WBC – white blood count

Table S2. Overall survival of all analyzed patients.

	All patients					
	No. of patients	OS [days]	95% CI	HR	95% CI	p-value
**Gender**						
Men	119	296	296 - nr	1.052	0.59 - 1.89	0.7
Women	69	nr	nr			
**Age**						
> 65	112	nr	nr	2.07	1.17 - 3.45	0.03
≤ 65	76	nr	296 - nr			
**Rai Stage**						
0	13	nr	19 -nr			0.52
1	40	370	nr			
2	69	296	296 - nr			
3	28	nr	nr			
4	32	nr	32 - nr			
**Binet stage**						
A	45	nr	nr			0.12
B	70	296	296 - nr			
C	29	nr	20 - nr			
**ECOG**						
2-4	46	nr	31 - nr	2.13	1.07 - 4.2	0.007
0-1	136	nr	296 - nr			
**WBC [×10⁹/L]**						
≤ 25	132	296	296 - nr	1.18	0.64 - 2.16	0.48
> 25	56	nr	nr			
**Hemoglobin [g/dL]**						
≤ 10	43	89	29 - nr	2.82	1.42 – 5.6	0.0005
> 10	144	nr	296 - nr			
**Platelets [×10⁹/L]**						
≤ 100	51	nr	25 - nr	2.69	1.36 - 5.29	0.0012
> 100	137	nr	296 - nr			
**Lactate dehydrogenase**						
Elevated	71	296	82 - nr	1.9	1.05 - 3.52	0.01
Normal range	90	nr	nr			
**BMI**						
<18,5	1	nr	nr			0.53
18,5-25	48	nr	nr			
25-30	87	nr	nr			
>30	36	296	33 - nr			
**Creatinine [mg/dL]**						
> 1.3	19	nr	26 - nr	1.29	0.47 - 3.72	0.35
≤ 1.3	163	nr	296 - nr			
**Deletion 17p**						
No	126	296	296 - nr	1.13	0.48 - 2.79	0.75
Yes	23	nr	nr			
**Deletion 11q23**						

Yes	21	nr	20 - nr	1.48	0.53 - 4.09	0.45
No	100	nr	296 - nr			
***TP53* mutation**						
Yes	24	296	8 - nr	1.51	0.54 - 4.21	0.37
No	106	nr	nr			
**CLL treatment status**						
Treatment naive	29	nr	nr			0.73
After	41	nr	nr			
During	117	nr	296 - nr			
**Lines of previous therapy**						
≥ 4	23	nr	100 - nr	1.17	0.5 - 2.77	0.79
0 – 3	164	nr	296 - nr			
**BTKi treatment**						
No	137	nr	nr	1.1	0.59 - 2.1	0.87
Yes	51	296	nr			
**Venetoclax treatment**						
Yes	36	nr	100 - nr	1.1	0.54 - 2.24	0.91
No	152	nr	296 - nr			
**Anti-CD20 treatment**						
Yes	46	nr	31 - nr	1.67	0.84 - 3.33	0.14
No	142	nr	296 - nr			

nr – not reached; BMI – body mass index; BTKi – Brutons tyrosine kinase inhibitor; ECOG – Eastern Cooperative Oncology Group; NA – not available; WBC – white blood count

Table S3. Characterization of treatment-naïve chronic lymphocytic leukemia patients diagnosed in the years 2010-2014 in the Institute of Hematology and Transfusion Medicine.

**Patient characteristics (n=138)**	
Median age, years (range)	64 (39-84)
Gender, n (%)	
Men	81 (58.6%)
Women	57 (41.4%)
Rai stage, n (%)	
0-2	113 (81.9%)
3-4	25 (18.1%)
WBC $\times 10^9$/L, median (range)	38.9 (9.9 – 755.5)
Hemoglobin g/dL, median g/dL (range)	12.9 (4.8 – 17.2)
Platelets $\times 10^9$/L, median G/L (range)	159 (6 – 431)
Observation time (months)	20.9 (3.1 – 138)

WBC – white blood count

Table S4. Association of analyzed factors with risk of hospitalization

	No		Yes		
**Gender**	N	(%)	N	(%)	p-value
Men	47	40.17%	70	59.83%	0.999
Women	27	39.71%	41	60.29%	
**Age**					
> 65	40	36.7%	69	63.3%	0.29
≤ 65	34	44.74%	42	55.26%	
**Rai Stage**					
0	5	38.46%	8	61.54%	0.36
1	17	42.5%	23	57.5%	
2	33	47.83%	36	52.17%	
3	11	42.31%	15	57.69%	
4	8	25.81%	23	74.19%	
**Binet stage**					
A	26	57.78%	19	42.22%	0.048
B	31	43.66%	40	56.34%	
C	8	28.57%	20	71.43%	
**ECOG**					
2-4	9	19.57%	37	80.43%	<0.001
0-1	63	47.01%	71	52.99%	
**WBC [×10⁹/L]**					
≤ 25	53	40.77%	77	59.23%	0.87
> 25	21	38.18%	34	61.82%	
**Hemoglobin [g/dL]**					
≤ 10	4	9.3%	39	90.7%	<0.0001
> 10	70	49.65%	71	50.35%	
**Platelets [×10⁹/L]**					
≤ 100	13	25,00%	39	75,00%	0.012
> 100	61	45.86%	72	54.14%	
**Lactate dehydrogenase**					
Elevated	21	29.58%	50	70.42%	0.009
Normal range	45	51.14%	43	48.86%	
**BMI**					
<18.5	0	0.00%	1	100,00%	0.76
18.5-24.9	21	44.68%	26	55.32%	
25-30	36	41.86%	50	58.14%	
>30	13	37.14%	22	62.86%	
**Creatinine [mg/dL]**					
> 1.3	5	26.32%	14	73.68%	0.32
≤ 1.3	65	40.63%	95	59.38%	
**Deletion 17p**					
No	8	36.36%	14	63.64%	0.64
Yes	55	43.65%	71	56.35%	
**Deletion 11q23**					
Yes	44	44.00%	56	56.00%	0.46

No	7	33.33%	14	66.67%	
***TP53* mutation**					
Yes	45	43.27%	59	56.73%	0.58
No	5	33.33%	10	66.67%	
**CLL treatment status**					
Treatment naive	14	48.28%	15	51.72%	0.46
After	17	43.59%	22	56.41%	
During	43	36.75%	74	63.25%	
**Lines of previous therapy**					
≥ 4	7	29.17%	17	70.83%	0.27
0 – 3	67	41.61%	94	58.39%	
**BTKi treatment**					
No	49	36.57%	85	63.43%	0.13
Yes	25	49.02%	26	50.98%	
**Venetoclax treatment**					
Yes	14	37.84%	23	62.16%	0.85
No	60	40.54%	88	59.46%	
**Anti-CD20 treatment**					
Yes	11	24.44%	34	75.56%	0.01
No	63	45,00%	77	55.00%	

BMI – body mass index; BTKi – Brutons tyrosine kinase inhibitor; ECOG – Eastern Cooperative Oncology Group; NA – not available; WBC – white blood count

Figure S1. Kaplan-Meier overall survival curves of the whole analyzed cohort depending on patient gender (a), age (b), Rai stage (c), Binet stage (d), ECOG status (e), white blood count (f), hemoglobin level (g), platelet level (h) lactate dehydrogenase level (i), body mass index (j), creatinine concentration (k), presence of 17p deletion (l), presence of 11q23 deletion (m), presence of *TP53* mutation (n), lines of previous CLL-directed treatment (o), treatment with a Brutons tyrosine kinase inhibitor (BTKi; p), treatment with venetoclax (r), treatment with anti-CD20 antibody (s) and CLL treatment status at COVID19 diagnosis (t). *p<0.05; **p<0.005; ***p<0.0005

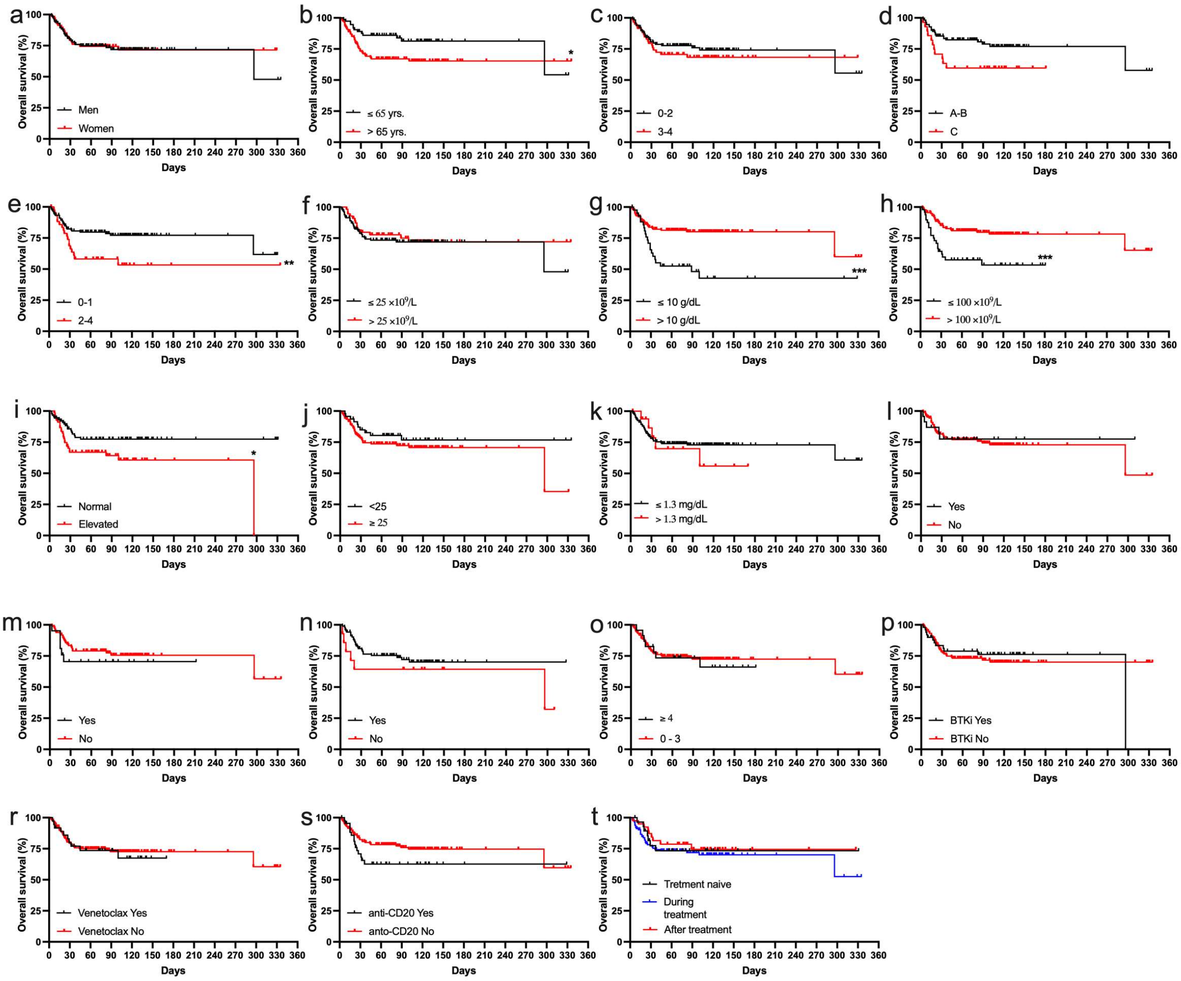

Figure S2. Kaplan-Meier overall survival curves of hospitalized patients depending on patient gender (a), age (b), Rai stage (c), Binet stage (d), ECOG status (e), white blood count (f), hemoglobin level (g), platelet level (h) lactate dehydrogenase level (i), body mass index (j), creatinine concentration (k), presence of 17p deletion (l), presence of 11q23 deletion (m), presence of *TP53* mutation (n), lines of previous CLL-directed treatment (o), treatment with a Brutons tyrosine kinase inhibitor (BTKi; p), treatment with venetoclax (r), treatment with anti-CD20 antibody (s) and CLL treatment status at COVID19 diagnosis (t). *p<0.05; **p<0.005; Log-rank test

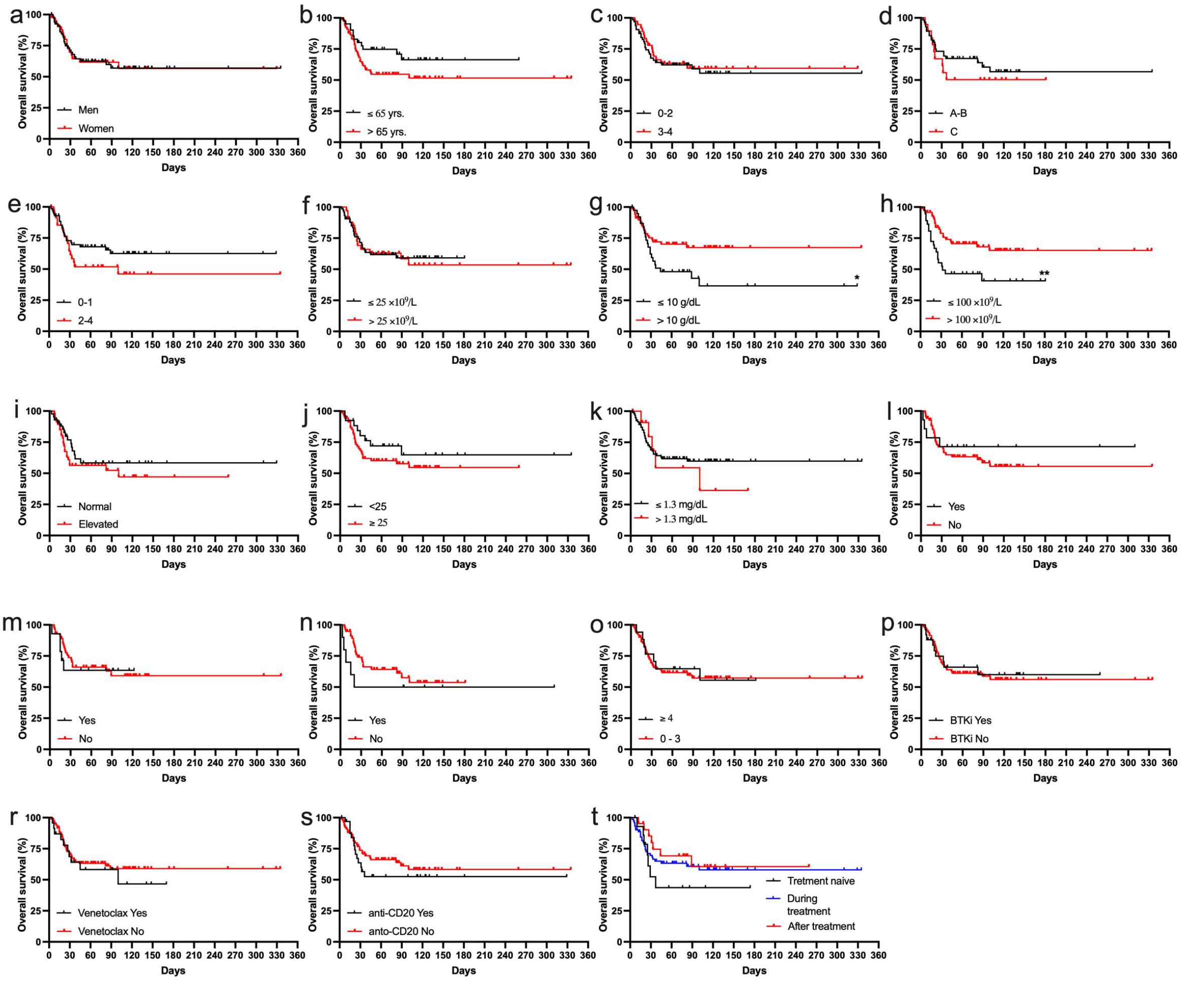

Supplement: Supplementary file 1 [file cancers-14-00558-s001.zip › COVID19 CLL supplementary information 22.01.2022.pdf]
